# Post-return of spontaneous circulation (ROSC) Accelerated Idioventricular Rhythm in the Setting of Severe Pancreatitis and Hyperkalemia

**DOI:** 10.7759/cureus.23573

**Published:** 2022-03-28

**Authors:** Alex Y Koo, Lei Gao

**Affiliations:** 1 Emergency Medicine, Madigan Army Medical Center, Tacoma, USA; 2 Cardiology, Highline Medical Center, Burien, USA

**Keywords:** ventricular rhythm, hyperkalemia, accelerated idioventricular rhythm, aivr, post-rosc ecg

## Abstract

A 73-year-old female with a history of coronary artery disease, hypertension, and diabetes presented to the emergency department in cardiac arrest. After cardiopulmonary resuscitation (CPR) and return of spontaneous circulation (ROSC), a post-ROSC electrocardiogram demonstrated Accelerated Idioventricular Rhythm (AIVR). The patient was found to have hyperkalemia due to anuric acute renal failure and antecedent severe pancreatitis. After medical management and dialysis, the patient recovered with good neurological recovery.

AIVR traditionally has been seen or documented as occurring after ischemia and subsequent coronary artery reperfusion. However, etiologies that promote ventricular automaticity must be considered as well. Electrolyte disturbances, drug toxicities such as digoxin, volatile anesthetics, cardiomyopathies, and ischemia can lead to AIVR. Treatment involves considering and correcting any underlying etiology with avoidance of antiarrhythmics, which may precipitate hemodynamic instability and asystole.

## Introduction

Acute pancreatitis is a common gastrointestinal disease, determined to be the most common gastrointestinal discharge diagnosis in the United States in 2009 and affecting 73.4 cases per 100,000 worldwide [1,2]. Inappropriate and premature activation of proteolytic enzymes induces local pancreatic tissue damage and activation of trypsinogen to trypsin. Trypsin subsequently leads to neutrophil migration and a cascade of proinflammatory cytokines. The proinflammatory cytokines can lead to increased endothelial permeability in various organs like the pancreas, lungs, kidneys, and intestines [3]. Acute kidney injury is a frequent and later complication of severe pancreatitis [4]. Earlier in the course of pancreatitis, increased endothelial permeability leads to decreased intravascular volume and hypovolemia. While acute kidney injury can occur at this stage, there are no morphological changes to the kidneys early on. As more pancreatic substances are released such as trypsin, bradykinin, and histamine, these substances have a direct toxic effect on the kidneys. Furthermore, proinflammatory cytokine activation leads to further kidney tubular ischemia [4,5]. Anuric renal failure portends dangerous electrolyte disturbances such as hyperkalemia. Subsequently, hyperkalemia can lead to a variety of arrhythmias, such as accelerated idioventricular rhythm (AIVR) [6,7].

AIVR is defined by three or more consecutive beats of an ectopic ventricular rhythm with rates from 50 to 120 beats/min [7,8]. AIVR occurs with increased vagal tone and decreased sympathetic tone. This leads to earlier phase 4 action potential depolarization within the myocardium and His-Purkinje fibers, which leads to faster cell depolarization. When the automaticity of an ectopic ventricular pacemaker overtakes the sinus node, AIVR becomes the predominant rhythm. The ectopic ventricular pacemaker can originate in the right or left ventricle leading to a left bundle branch and right bundle branch morphology, respectively.

Accelerated Idioventricular Ventricular Rhythm (AIVR) is traditionally taught as a transient rhythm after coronary artery reperfusion, but etiologies that promote ventricular automaticity must be considered as well. Other etiologies, such as electrolyte disturbances, drug toxicities as digoxin, volatile anesthetics, cardiomyopathies, and ischemia, can lead to AIVR [8,9]. This case highlights an instance of hyperkalemia-induced AIVR with precipitating factors of severe acute pancreatitis with anuric renal failure.

## Case presentation

**Figure 1:**
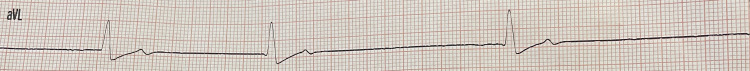
Initial pulseless electrical activity rhythm, demonstrating a wide complex, irregular rhythm

A 73-year-old female with a history of coronary artery disease, hypertension, and diabetes presented to the emergency department in cardiac arrest. Per emergency medical services (EMS), she became unresponsive during breakfast at home with her son. Cardiopulmonary resuscitation (CPR) was initiated by her son and EMS arrived on the scene to find the patient in pulseless electrical activity (PEA) with an initial rhythm strip obtained (Figure 1).

**Figure 2:**
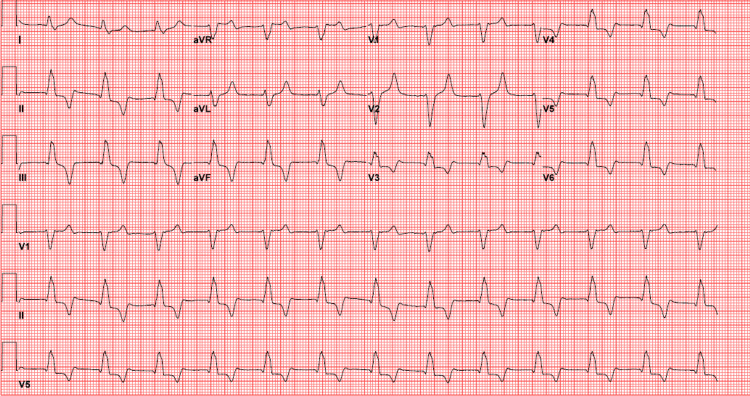
Post-return of spontaneous circulation electrocardiogram showing a regular wide-complex rhythm with no discernible P waves. The rate is 77 beats/min. The axis is normal. There is left bundle branch block morphology with narrow-based, tall T waves.

The patient received 24 minutes of CPR and three doses of epinephrine with the return of spontaneous circulation (ROSC). A post-ROSC electrocardiogram (ECG) was obtained in the emergency department (Figure 2).

The ECG was interpreted as an AIVR with peaked T waves. Subsequent history with the patient’s son revealed that the patient had progressive nausea, vomiting, and epigastric pain over the previous week. She complained of anuria for two days prior to her cardiac arrest.

Lab analysis revealed a new glomerular filtration rate of 4 milliliters per minute (mL/min/1.73m^2^), creatinine of 9.6 milligrams per deciliter (mg/dL) (reference range: 0.5-1.2 mg/dL) with potassium of 6.9 millimoles per liter (mmol/L) (reference range: 3.5-5.0 mmol/L). In addition, the patient had significant leukocytosis, hyponatremia, lipase elevation, transaminitis, and lactic metabolic acidosis with a pH of 6.9 (reference range: 7.35-7.45). Previous labs obtained by her primary care physician two weeks prior had been normal.

**Figure 3:**
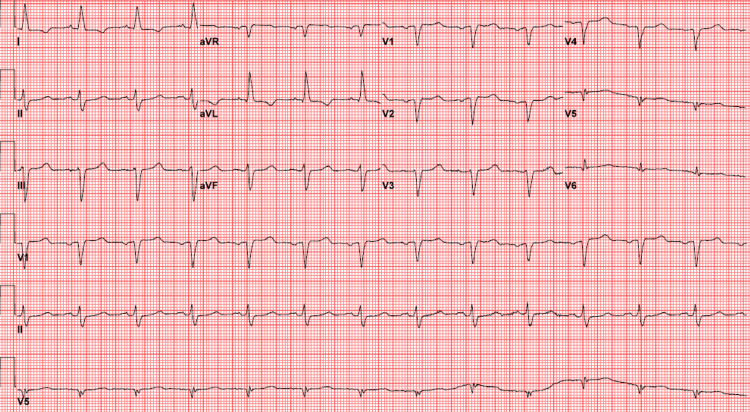
Post-dialysis electrocardiogram showing normal sinus rhythm and resolution of peaked T waves. Findings of normal sinus rhythm and a rate of 78 beats/min. Poor R-wave progression. Left axis deviation with T-wave inversions in lateral leads I, aVL. Similar to her ECG one year prior.

The patient subsequently received calcium gluconate, insulin and dextrose, albuterol, empiric antibiotics, and sodium bicarbonate. She underwent a noncontrast computed tomography (CT) of her abdomen. On CT, there was diffuse peripancreatic stranding and pancreatic enlargement without fluid collections, consistent with pancreatitis. The patient was admitted to the intensive care unit (ICU) with a diagnosis of severe acute pancreatitis with acute renal failure. The patient underwent emergent dialysis and recovery of kidney function. A post-dialysis ECG was obtained with the resolution of peaked T waves (Figure 3).

During her hospital course, etiologies of her pancreatitis were considered. With a normal abdominal ultrasound without cholelithiasis, common bile duct dilation, unremarkable triglycerides, no recent medication changes or alcohol history, the cause of her pancreatitis remains idiopathic. The patient was discharged on hospital day 10 to a skilled nursing facility with full neurological recovery, voiding with a creatinine of 0.91 mg/dL, and resolution of her electrolyte derangements. 

## Discussion

Pancreatitis can manifest in local complications of peripancreatic fluid collections and necrosis to systemic complications of multi-organ failure. Diagnosis of pancreatitis is established through at least two of the following three criteria: a) abdominal pain consistent with the disease, b) lipase and/or amylase elevation three times the upper limit of normal, and c) characteristic findings on abdominal imaging. In this case, the patient had all three criteria to establish a diagnosis of pancreatitis. Pancreatitis severity is a spectrum and can be graded based on local and systemic complications and the timeframe of complications. Severe pancreatitis occurs in about 20% of cases of acute pancreatitis. It is defined by persistent organ failure greater than 48 hours by the modified Marshall Score, which is calculated based on respiratory, renal, and cardiovascular parameters [1,10]. The patient had elements of anuric renal failure and cardiovascular collapse, a response to the systemic inflammatory response of pancreatitis. Without the ability to excrete potassium, the patient developed hyperkalemia with a rhythm of AIVR.

AIVR traditionally has been identified as occurring after ischemia and subsequent coronary artery reperfusion, but in the emergency department, observation of AIVR is becoming less common given the increasing prevalence of percutaneous coronary intervention (PCI) over thrombolytic therapy [9,11,12]. A broad differential must always be considered when encountering AIVR. Treatment involves considering and correcting any underlying etiology with avoidance of antiarrhythmics, which may precipitate hemodynamic instability and asystole. In this case, her post-ROSC ECG of AIVR has an initial differential for ischemia and spontaneous reperfusion, hyperkalemia, or post-ROSC AIVR. Given the LBBB morphology of AIVR, Sgarbossa or Smith Modified Sgarbossa Rule can be considered to evaluate for occlusion myocardial infarction; however, there are no validated studies of Sgarbossa for AIVR. In this patient’s case, Sgarbossa criteria were negative with serial troponins and echocardiogram excluding a persistent myocardial infarction. A transient occlusive myocardial infarction and spontaneous coronary reperfusion could be considered, but it was determined to be unlikely. The patient’s work-up did not reveal a transient clinical history of chest pain and subsequent ECGs did not have any new T-wave abnormalities or evidence of Wellens Syndrome [13]. In addition, there was a more likely diagnosis of severe pancreatitis with metabolic acidosis and hyperkalemia. It is contributory that both her hyperkalemia and her severe pancreatitis with acidosis contributed to her post-ROSC AIVR.

What do we already know about this clinical entity?

AIVR is defined by a monomorphic, ventricular ectopic rhythm of rates 50-120 beats/min.

What is the major impact of the image(s)?

While sometimes a benign rhythm after reperfusion, post-ROSC, or in athletic hearts, AIVR can also have underlying etiologies of electrolyte disturbances, drug toxicities, and ischemia. Treatment of AIVR should focus on identifying and treating the underlying condition. Antiarrhythmics should be avoided as they can precipitate asystole.

How might this improve emergency medicine practice?

This case demonstrates the differential that must be considered when encountering AIVR, which can help early diagnosis and treatment.

## Conclusions

AIVR is considered a benign rhythm after reperfusion, post-ROSC, or in athletic hearts, but AIVR can also have underlying etiologies of electrolyte disturbances, drug toxicities, and ischemia. With severe pancreatitis, anuric renal failure can lead to significant hyperkalemia and metabolic acidosis, precipitating AIVR. Treatment of AIVR should focus on identifying and treating the underlying condition.
